# A metagenetic approach to determine the diversity and distribution of cyst nematodes at the level of the country, the field and the individual

**DOI:** 10.1111/mec.13434

**Published:** 2015-11-26

**Authors:** Sebastian Eves‐van den Akker, Catherine J. Lilley, Alex Reid, Jon Pickup, Eric Anderson, Peter J.A. Cock, Mark Blaxter, Peter E. Urwin, John T. Jones, Vivian C. Blok

**Affiliations:** ^1^ Division of Plant SciencesCollege of Life SciencesUniversity of DundeeDundeeDD1 5EHUK; ^2^ Centre for Plant SciencesUniversity of LeedsLeedsLS2 9JTUK; ^3^ Science and Advice for Scottish AgricultureEdinburghEH12 9FJUK; ^4^ Scottish Agronomy LtdArlary FarmMilnathortKinrossKY13 9SJUK; ^5^ Information and Computational Sciences GroupDundee Effector ConsortiumThe James Hutton InstituteInvergowrieDundeeDD2 5DAUK; ^6^ Institute of Evolutionary BiologyUniversity of EdinburghEdinburghEH8 9YLUK; ^7^ Cell and Molecular Sciences GroupDundee Effector ConsortiumThe James Hutton InstituteInvergowrieDundeeDD2 5DAUK; ^8^ School of BiologyUniversity of St AndrewsNorth HaughSt AndrewsKY16 9TZUK

**Keywords:** distribution, diversity, metagenetics, mitotype, potato cyst nematode

## Abstract

Distinct populations of the potato cyst nematode (PCN) *Globodera pallida* exist in the UK that differ in their ability to overcome various sources of resistance. An efficient method for distinguishing between populations would enable pathogen‐informed cultivar choice in the field. Science and Advice for Scottish Agriculture (SASA) annually undertake national DNA diagnostic tests to determine the presence of PCN in potato seed and ware land by extracting DNA from soil floats. These DNA samples provide a unique resource for monitoring the distribution of PCN and further interrogation of the diversity within species.

We identify a region of mitochondrial DNA descriptive of three main groups of *G. pallida* present in the UK and adopt a metagenetic approach to the sequencing and analysis of all SASA samples simultaneously. Using this approach, we describe the distribution of *G. pallida* mitotypes across Scotland with field‐scale resolution. Most fields contain a single mitotype, one‐fifth contain a mix of mitotypes, and less than 3% contain all three mitotypes. Within mixed fields, we were able to quantify the relative abundance of each mitotype across an order of magnitude. Local areas within mixed fields are dominated by certain mitotypes and indicate towards a complex underlying ‘pathoscape’. Finally, we assess mitotype distribution at the level of the individual cyst and provide evidence of ‘hybrids’. This study provides a method for accurate, quantitative and high‐throughput typing of up to one thousand fields simultaneously, while revealing novel insights into the national genetic variability of an economically important plant parasite.

## Introduction

Cyst nematodes are microscopic obligate root parasites that cause significant economic damage; predicted global costs associated with yield loss and control measures extend into the billions of US dollars. Cyst nematodes pose a significant challenge to modern agricultural practice due to a combination of their unusual biology and the absence of effective control measures for many species (Nicol *et al*. 2011).

The juvenile potato cyst nematode (PCN), *Globodera* spp., invades host roots, establishes a feeding site and develops into a mature female or male. Females remain sedentary, while males leave the root and migrate to locate a female. Females are polyandrous and each may mate with multiple males. Once fertilized, the female's body dries to encase the eggs in a hard cyst. Nematodes within eggs inside the cysts can remain dormant in the soil for up to 20 years (Turner 1996) and may be dispersed by wind, land cultivation, human travel and even crops. As a result, cyst nematodes are easily spread across long distances. PCN is present on both EPPO and USDA quarantine organism lists.

Two species of PCN are of agronomic importance in many temperate potato growing regions: *G. rostochiensis* and *G. pallida*. Wide‐scale deployment of the H1 resistance gene in the UK has been highly effective in controlling the former (Barone *et al*. 1990). However, this has led to strong selection of *G. pallida*, which has subsequently increased in prevalence and is now present in approx. 92% of the potato fields in England and Wales infested with PCN (Minnis *et al*. 2002). The spread of *G. pallida* is of concern for the protection of seed potato land in Scotland. Development of effective resistance to *G. pallida* has been challenging and commercially acceptable potato varieties with high levels of resistance have only recently become available. The performance of these varieties in the field with regard to suppressing *G. pallida* and in terms of other traits is still under evaluation.

Despite the fact that the genetic variation of both species in Europe reflects only a small component of the total diversity that was introduced from South America (Plantard *et al*. 2008), it is hypothesized that this disparity between species with regard to the availability of host resistance reflects their respective genetic variation. For example, a recently identified family of parasitism genes contained substantially more variation in UK *G. pallida* populations compared to *G. rostochiensis* (Eves‐van den Akker *et al*. 2014). Consistent with this, multiple genetically distinct populations of *G. pallida* have been characterized in the UK (Kort *et al*. 1977; Phillips & Trudgill 1998; Hockland *et al*. 2012). Furthermore, *G. pallida* pathotypes exist which differ in their ability to overcome various sources of resistance and therefore cultivar choice can be critical. Even prior to the recent restrictions in nematicide applications (EU Regulation EC 1107/2009), one of the most effective control measures for PCN was host resistance. Thus, recent work has focused on identifying rapid and accurate means to distinguish PCN populations to ultimately assist in cultivar choice (Hoolahan *et al*. 2012; Mimee *et al*. 2015).

In this vein, a mitochondrial gene, cytochrome B (cyt B), has been used for phylogenetic inference of three genetically distinct groups of *G. pallida* consistent with the three main introductions into Europe (Plantard *et al*. 2008). Clades Ic2, 3 and 6 contain European populations with phenotypes of pathotype 1, 2 and 3 (Hockland *et al*. 2012). While clearly not the causal mutations underlying differences in pathogenicity, these three ‘mitotypes’ represent a promising opportunity to study the diversity of *G. pallida* in the UK and to examine their distributions.

Science and Advice for Scottish Agriculture (SASA) carry out annual preplant PCN tests of seed potato land and, following the implementation of EU Directive 2007/33/EC, have also carried out an annual random survey of 0.5 % of ware land for PCN since 2010 (Reid *et al*. 2015). Between 80 and 300, soil cores across each field are collected and pooled (https://www.sasa.gov.uk/sites/default/files/PCN%20Booklet%202014.pdf). DNA is extracted from sample float material, which contains nematode cysts, and independent qPCR‐based assays are used to estimate the abundance of either *G. rostochiensis* or *G. pallida*. These pre‐existing SASA samples provide a unique resource for monitoring the distribution of PCN and for further interrogation of the diversity within the species.

The use of cyt B as a proxy for distinct *G. pallida* groups, in conjunction with both a unique DNA sample catalogue and the advent of massively parallel next‐generation sequencing, allows questions to be posed that were previously technically unfeasible. We describe a metagenetic approach to determine the presence, and quantify the relative abundance, of each *G. pallida* mitotype in ~800 samples, covering 687 fields across potato growing regions of Scotland, in a single high‐throughput experiment. The approach described is broadly applicable to many pathosystems and has revealed novel insights into the complexity of PCN genetic diversity at the level of the country, the field and the individual animal.

## Methods

### DNA samples

The mitotype diversity was studied in 687 fields (sampled between 2011 and 2014), 64 samples taken at regular intervals across two field sites, 22 individual cysts and various control mixes described in the relevant section.

Individual field samples were collected by SASA as part of annual surveys (https://www.sasa.gov.uk/sites/default/files/PCN%20Booklet%202014.pdf). In brief; each sample is a pool of either 80 or 300 individual five millilitre cores per hectare (depending on the outcome of previous tests or growing seasons), collected from a ‘W’‐shaped transect across each area. Samples were processed for DNA extraction and PCN detection as described (Reid *et al*. 2015). Each sample (standard 1500 ml/ha or reduced 400 ml/ha) was dried at 37 °C for a minimum of 2 days, and an automated MEKU nematode carousel was used to collect the float material into 200‐μm sieves. DNA was extracted from up to 2 ml of float material (which contains any cysts) using a TissueLyser II with 2 × 24 adapter set (QIAGEN) and MagMAX^™^ Express‐96 Deep Well Magnetic Particle Processor (Life Technologies). DNA samples were tested for the presence of PCN using a qPCR‐based method (Reid *et al*. 2015). PCN‐positive samples (~200 per species per year) were collated into individual plates and archived at −20 °C. In addition, a grid sampling approach was employed across two regions of a field (OS Ref: NT 484 810), either 15 by 10 m squares or 5 by 16 m squares. Float material from each position in the grid was prepared separately as described above.

### Primer and barcode design

While cyt B has been used for phylogenetic inference of *G. pallida* populations (Plantard *et al*. 2008), a specific region amenable to the strict requirements of a metagenetic approach had not been identified. A region, less than 450 base pairs (bp), which is descriptive of each of the three mitotypes by at least two SNPs in a total of 5 polymorphic positions and is flanked by highly conserved regions for primer binding, was identified from 66 available cyt B sequences (Dryad Accession doi: 10.5061/dryad.pd7r6). Several different primer combinations, all specific to *G. pallida,* were explored, the pair which produced the most reliable amplification at low template concentrations (F1, R1) was selected for further study (Table S1, Supporting information). With respect to GenBank DQ631912.1, this primer pair amplifies the region 281–590 bp from the start codon.

To distinguish between ~800 samples postsequencing, each sample was amplified with a unique identifier or barcode appended to the primer. Synthesis of 900 unique primers is prohibitively expensive. However, pairwise combinations of 30 unique forward and 30 unique reverse primers were used to generate 900 unique pairs. In the first instance, unique primers were generated by fusion to Illumina adapters and barcode sequences. The reduction in amplification efficiency attributed to the 55‐bp overhang was prohibitive for the low template concentration intrinsic to SASA samples. Thus, 30 unique 4‐bp barcodes were designed, each at least two base pairs different from any other (Table S2, Supporting information). For each unique 4‐bp barcode, forward and reverse oligonucleotide primers were synthesized with two 5′ adenosines, followed by the relevant barcode, and finally the primer sequence. There was no observable detrimental effect of these 6‐bp overhangs on amplification efficiency (not shown).

Given that every pairwise combination of primers was mixed to generate 900 unique pairs, cross‐contamination between successive synthesis reactions would drastically affect the validity of the approach. Additional precautions can be requested from certain companies, but at a vastly increased cost. For the number of primers required, these additional costs restrict the scope for wide adoption and regular use of the approach. Here, standard primer synthesis reactions were commissioned, however, one primer was ordered per day from three different companies for 20 days. This ensures that other unrelated reactions will intersperse the metagenetic primers. We accept that metagenetic primers will contain traces of other primers, but it was deemed unlikely they would target the same species, gene, region of gene, and utilize the novel barcode structure. This reduced the cost for this aspect of the project by ~20 fold.

### PCR, library preparation and sequencing

For all liquid handling steps prior to PCR, filter tips were used to minimize cross‐contamination from aerosols. All PCRs were carried out using Phusion Hi‐fidelity proofreading polymerase following the manufacturer's instructions (NEB), with 5 μl of template. Thirty seven cycles of 20 s at 98 °C, 30 s at 64 °C and 60 s at 72 °C were followed by a final extension of 5 min at 72 °C. All PCRs were analysed by gel electrophoresis and assigned to two groups. One microlitre of the high amplification group or 10 μl of the low amplification were pooled to ensure sufficient read depth per sample. Total pooled PCR products (2–3 ml) were precipitated at −80 °C overnight with the addition of 1 volume isopropanol and 0.2 volumes 3 m sodium acetate. Precipitated DNA was pelleted by centrifugation at 13 000 *g* for 20 min at 4 °C. The pellet was washed once in 70% ethanol, allowed to air dry, and resuspended in 180 μl of 10 mm Tris‐Cl, pH 8.5. Concentrated PCR products were additionally purified by size selection using MagJET NGS Cleanup and Size Selection beads (Thermo Scientific) following the manufacturer's instructions for adapter removal for an amplicon of 300 bp. Size selected blunt ended DNA was eluted from the beads in 50 μL and analysed by gel electrophoresis. Library preparation was carried out by Edinburgh Genomics (University of Edinburgh, Edinburgh, UK) using an Illumina TruSeq PCR Free kit following the manufacturer's instructions but omitting the shearing step prior to blunt‐end adapter ligation. The library was sequenced on a MiSeq instrument using 300 base paired‐end reads (v3 chemistry) and 30% PhiX spike‐in.

### Bioinformatic analyses

All custom python scripts and detailed protocols are available under GitHub repository https://github.com/sebastianevda/SEvdA_metagen.git. In brief, raw reads were trimmed of leading and trailing low‐quality bases (Phred < 30), followed by a 10‐bp sliding window of average Phred score <30, followed by a minimum length restriction of 150 bases using Trimmomatic (Bolger *et al*. 2014). Overlapping pairs of reads were assembled using Pear (Zhang *et al*. 2014), converted to Phred 64 coding using Trimmomatic, and subsequently converted into FASTA format using the fastx toolkit (Gordon & Hannon 2010). All sequences were prepared in the same orientation and those which contained the forward and reverse primer binding sites were retained for further analysis. Custom python script 1 was used to trim remaining adenosines 5′ to the barcode site, trim the primer binding site, impose a minimum length restriction of 305 bp and return a tabular output in the format, barcode 1 barcode 2 amplicon. The number of unique amplicons per barcode pair was counted (count_unique_seq_per_barcode_pair.sh) and those amplicons which contributed to less than 5% of the total for each barcode pair were identified using custom python script 2 and removed from further analysis.

### Data analysis

The frequency of all remaining amplicons was counted, and the 50 most common were aligned and refined using MUSCLE (Edgar 2004). The alignment was used to construct a Bayesian phylogeny with 2 million generations. Model selection (F81) and molecular phylogenetic construction was carried out using TOPALi (Milne *et al*. 2009). The phylogenetic tree was mid‐point rerouted and formatted in figtree v1.4 (http://tree.bio.ed.ac.uk/software/figtree/). For each barcode pair, the number of reads corresponding to each of the mitotypes from the phylogeny was counted using custom grep scripts (see list of commands), and the relative contribution to the total was calculated in Excel. An integer cut‐off for each mitotype was set at the maximum number of reads observed in barcode pairs which were known not to contain that mitotype. Relative abundance plots on maps or 3D surface plots were generated using custom python scripts 3a/b and 4a/b, respectively, in the Canopy wrapper for Cartopy and matplotlib (enthought.com/products/canopy).

## Results

Thirty unique barcodes were designed, 4 base pairs in length, with at least 2 base pairs mismatch between any two (Table S2, Supporting information). Thirty forward and 30 reverse oligonucleotide primers were synthesized, each within a set having a different 5′ barcode. Due to exponential scaling, every pairwise combination of primers gives rise to 900 unique pairs. Each unique pair was used to amplify a 310‐bp region of cyt B from one sample only. The result is that PCR products can be pooled, sequenced in parallel and subsequently deconvoluted into their original samples bioinformatically based on the barcode sequence.

### Amplification and sequencing

Samples included various levels of resolution from fields across a country (687), two grid samples across a field (64), a range of individual cysts (22) and various controls (35). All PCRs were pooled approximately quantitatively prior to sequencing to ensure adequate read depth of each. Each 310‐bp amplicon was sequenced almost in its entirety from both ends to generate overlapping 300‐bp paired‐end reads. From an initial population of ~12 million reads, 60% (7 076 131) remained with a minimum length of 150 bp after trimming of adapter sequences and low‐quality bases (Phred <q30). Following trimming, overlapping paired‐end reads were recapitulated into the full‐length cyt B amplicon with >98% success rate. Only sequences which contained both the forward and reverse primer site used to generate the amplicon, one barcode on each end, and that had a minimum length of 305 bp were retained for further analysis (6 614 854). Not including the barcode sequences, the 6 614 854 reads comprised 46 503 unique amplicons – vastly more than anticipated – the majority of which occurred only once.

### Sensitivity

**Figure 1:**
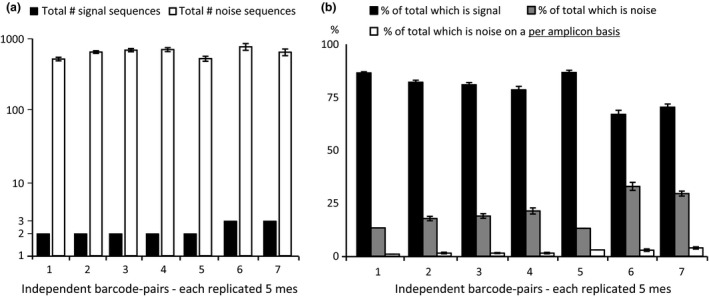
Sensitivity. Seven different control conditions (each replicated with five unique barcode pairs) contained mixes of either two or three plasmids encoding known cyt B sequences. (a) The number of unique signal sequences (black) compared to the number of unique noise sequences (white). (b) The contribution of unique sequences to signal (black), noise (grey) or noise on a per unique sequence basis (white). Removing all unique sequences which contribute <5% to the total number of sequences for that barcode pair efficiently removes all noise from the control samples.

A range of controls was used to determine the sensitivity of the approach. Seven different control amplifications (each replicated with 5 unique barcode pairs) contained template mixes of either two or three plasmids encoding known cyt B sequences. For each control condition, reads corresponding to the plasmid sequences with 100% identity were considered signal, while all other reads were considered noise. Approximately 1000 unique sequences corresponding to noise were consistently identified for each barcode pair (Fig. 1a). The cumulative noise contribution can be up to 35% of the total number of reads (Fig. 1b). However, the vast majority of the ~1000 noise sequences occurred only once, and none of them individually contributed to more than 5% of the total reads for a given barcode pair (Fig. 1b). Thus, there was a large amount of effectively random, low‐frequency noise, rather than high‐frequency contamination by a particular sequence. By removing all unique sequences which contributed <5% to the total number of sequences for that barcode pair, we could efficiently remove all noise from the control samples. A 5% cut‐off was imposed for all other barcode pairs based on the above controls. This determined the sensitivity of the approach, such that if a genuine sequence in the population is present below 5%, it will not be detected.

The percentage cut‐off efficiently removed most noise from the system. However, if the total number of reads per barcode pair is low, the percentage contribution of noise to the total can exceed 5% and would therefore wrongly be retained as true signal. Thus, spare barcode pairs which were never used in the experiment, but which do contain low numbers of reads, are critical to remove remaining noise. These cases typically consist of low total numbers of reads (hundreds rather than thousands) and result from either cross‐contamination or sequencing/PCR error on the barcode. The maximum read number observed for any unused barcode pair which should not contain any reads was used as an integer cut‐off, below which all reads were discarded on a per mitotype basis. A total of approximately 5.6 million amplicons remained after percentage cut‐off followed by integer cut‐off, with an average of ~10 000 reads per unique barcode pair.

### Specificity

**Figure 2:**
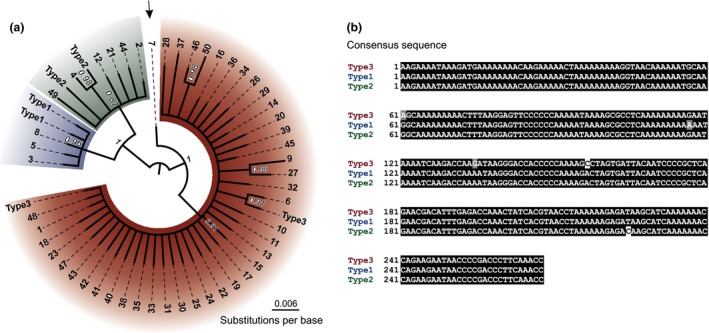
Bayesian phylogeny of the 50 most common sequences. (a) The 50 most common sequences (numbered in order of frequency) were used to construct a Bayesian phylogeny with 2 million iterations. All sequences were subdivided into three Clades, each corresponding to one of the known mitotypes, with the exception of the 7th most common sequence. Sequence 7 contains all SNPs descriptive of mitotype 3, with the exception of position 61, normally descriptive of mitotypes 1 and 2. (b) The consensus sequence in each clade corresponds with 100% identity to the descriptive SNPs of each mitotype.

The combination of cut‐off procedures drastically reduced the number of unique amplicons from 46 000 to 2 060. To determine the specificity of the approach, the 50 most common amplicons (numbered in order of frequency) were used to construct a Bayesian phylogeny, that efficiently separated them into three major clades (Fig. 2a). The consensus sequence in each clade corresponds with 100% identity to one of the three known mitotypes (Fig. 2b), giving confidence in the approach. These 50 most common amplicons represent just 2.5% of the unique sequences, but account for 99.63% of the total number of reads postcleaning. For each barcode pair, the numbers of sequences corresponding with 100% identity to any sequence in clades 1, 2 or 3 only were counted, resulting in a relative frequency of each mitotype for that sample. The 7th most common sequence is similar to mitotype 3, as seen from its position in the phylogeny and sequence. Sequence 7 contains all SNPs descriptive of mitotype 3, with the exception of position 61, normally descriptive of mitotypes 1 and 2 (Table S3, Supporting information).

**Figure 3:**
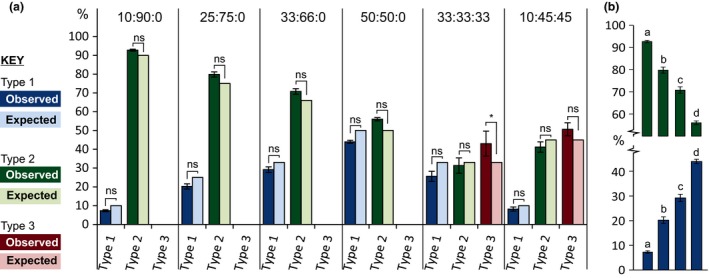
Specificity. Ratios of plasmids containing known sequences were mixed and analysed in parallel with test samples. (a) Observed ratios (dark bars) are not significantly different (ns) from the expected ratios (light bars) with the exception of mitotype 3 only in the 33:33:33 mix (χ^2^ *P* value cut‐off 0.05), suggesting the relative abundance of reads corresponding to each mitotype is a faithful reproduction of the ratio of mitotypes in the original sample. (b) While the mitotypes 1 and 2 ratios do not differ from expected values, they do differ between samples (Student's *t*‐test with Bonferroni correction for multiple tests, *P* < 1E^−5^), suggesting we can specifically distinguish, for example, between 10% of mitotype 1 and 25% of mitotype 1.

In addition, known ratios of either two or three mitotypes (encoded by plasmids) were mixed and used for PCR amplification alongside field samples, each with 5 technical replicates. Fig. 3 describes the expected and observed percentages in terms of mitotype 1: mitotype 2: mitotype 3. For a range of ‘two mitotype’ mixes within an order of magnitude (10:90, 25:75, 33:66, 50:50), and even complex ‘three mitotype’ mixes across a similar range (10:45:45 and 33:33:33), observed ratios do not differ significantly from expected ratios, with the exception of mitotype 3 only in the 33:33:33 mix (χ^2^ *P* value cut‐off 0.05, Fig. 3a). This suggests the ratio of reads corresponding to each mitotype within a barcode pair is a faithful reproduction of the ratio of mitotypes in the original sample. Furthermore, comparison of one mitotype between mixes shows that 10% is significantly different from 25%, and indeed 33% and 50% (Student's *t*‐test with Bonferroni correction for multiple tests, *P* < 1E^−5^, Fig. 3b). Together, these data show that the approach used here can be used to infer frequency of mitotypes within and between samples.

### The distributions of *G. pallida* mitotypes at country‐scale resolution

**Figure 4:**
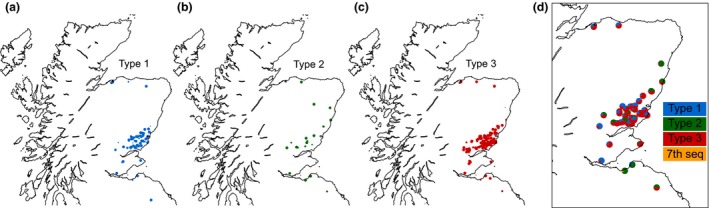
The national distribution of *G. pallida* mitotypes: relative abundance at each site. (a–c) The presence of mitotypes 1, 2 and 3 across Scotland's potato growing regions is indicated by blue, green or red markers, respectively. The size of each marker is scaled by the relative abundance of the relevant mitotype within each field. (d) Markers plotted as pie charts better compare the relative abundance in mixed fields.

During the sampling procedure, several soil cores from a range of loci across a given field are collected and pooled to build a complete picture of the PCN diversity in a single mixed DNA sample. With the high stringency approach, we were able to detect *G. pallida* in ~76% of fields previously identified as ‘*G. pallida* positive’ by qPCR (Reid *et al*. 2015). Of the fields, ~79% contain a single mitotype, ~18% contain a mix of two mitotypes, and ~2.3% contain a mix of all three mitotypes. Given that a single barcode pair was used per sample, and the location of the field was recorded upon sampling, the national distribution of each mitotype can be plotted (Fig. 4a–c). As is clear from Fig. 4, all three mitotypes are broadly prevalent across the majority of Scotland. Mitotype 3 dominates and contains the most sequence variability (Fig. 2), while mitotype 2 is the least prevalent yet contains more variation in sequence than mitotype 1. Interestingly, mitotypes 1 and 3 are approximately twice as likely to co‐occur as 1 and 2, or 2 and 3 (even when scaled by total occurrences). The 7th most common sequence only occurs in a single field and is present at 8% relative to 92% mitotype 2. For mixed fields only, the relative abundance of each mitotype in each field can be plotted where the size of the segment scales by the relative abundance of each mitotype (Fig. 4d). This identifies well those fields which contain more than one mitotype and indeed the relative abundance of each mitotype within that field. However, due to the inclusive sampling approach across each field, this does not intrinsically inform as to the distribution of mitotypes within a field.

### The distribution of *G. pallida* mitotypes at field‐scale resolution

**Figure 5:**
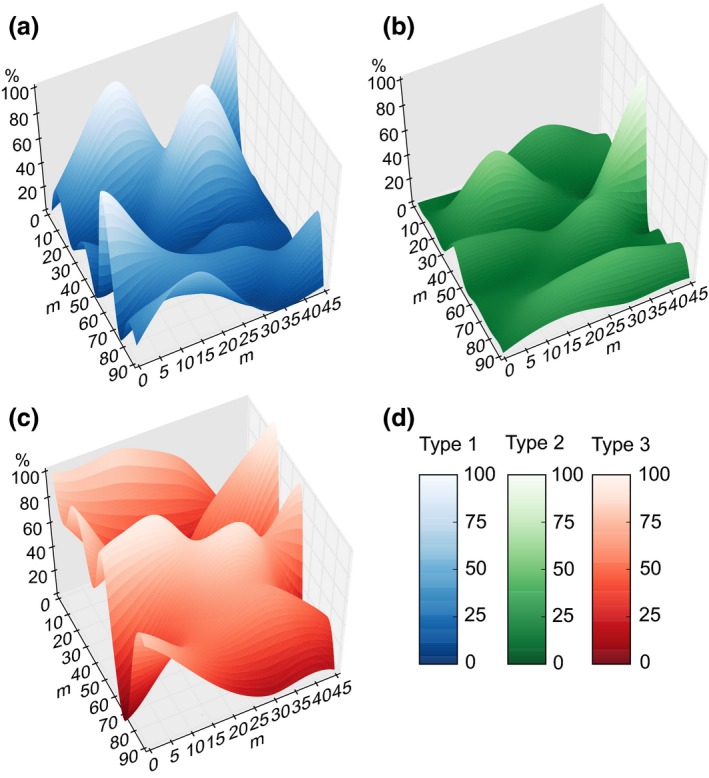
Mapping of the distribution of mitotypes within a mixed field yields complex ‘Pathoscapes’. A series of samples taken at 15 by 10 m squares across a field were prepared separately and each assigned their own barcode pair. The relative abundance of each mitotype at each locus in the field may reflect a complex underlying ‘pathoscape’. Highly localized peaks of primarily single mitotypes dominate.

A grid sampling approach was employed across two fields to determine the distribution of mitotypes within mixed fields. Each sample was prepared separately and assigned its own barcode pair. Thus, the distribution of mitotypes across the field can be plotted where the X and Y axes correspond to the particular coordinates within a field, and the Z axis represents the relative abundance of each mitotype (Fig. 5). Mixed fields do not contain an even distribution of mitotypes; local peaks and troughs of single mitotypes dominate and may reflect a complex underlying ‘pathoscape’ within mixed fields. Furthermore, we were able to replicate this complex pattern at an additional site (Fig. S1, Supporting information), albeit with a drastically different composition. This highlights the importance of sampling multiple cores from a single field as described above (80–300 per hectare) for analysing national distributions.

### The distribution of *G. pallida* mitotypes within individual cysts

**Figure 6:**
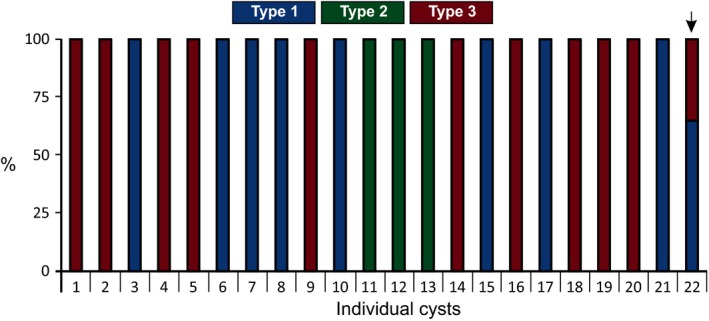
The distribution of mitotypes at the level of individual cysts. The genetic complement of 22 individual cysts, collected from mixed and single mitotype fields, with respect to the mitotype structure was analysed. The vast majority of cysts comprise only a single mitotype. However, cyst 22 (arrow) appears to contain a mix of mitotype 1: mitotype 3 at a ratio of approximately 33:66 – well within the confidence limits of the experiment.

The level of resolution can be extended to allow analysis of single cysts. DNA extracted from 22 individual cysts was included in the PCRs, each with its own barcode pair. The vast majority of single cysts tested contained DNA of a single mitotype (Fig. 6). However, cyst 22 appears to contain a mix of mitotypes 1 and 3. Given that (i) cyst nematodes are polyandrous; (ii) multiple mitotypes co‐occur within fields; (iii) cysts contain hundreds of eggs, each of which is an individual animal; and (iv) the 33:66 distribution is well within our confidence limits, and cyst 22 is designated as a putative ‘hybrid’. We are unable to determine whether hybrid cysts contain a mixture of homogenous and/or heterogeneous individuals.

## Discussion

Here we demonstrate the power of a metagenetic approach to simultaneously describe the genetic diversity of an economically important plant parasite at three levels of resolution: country, field and individual animal.

The unusual biology of cyst nematodes introduces complexity into the above scenario. While the polymorphisms in the cyt B show a relationship with populations that differ in their virulence, they are presumably not the causal agent. This is particularly important given the, albeit rare, precedent for paternal inheritance of mitochondrial DNA (Hoolahan *et al*. 2011) and the multipartite mitochondrial genome of *G. pallida* with associated gene duplications (Gibson *et al*. 2007). The region of cyt B used in the present study contains an A/T polymorphism which distinguishes between the copies present on circles 1 and 3 (Armstrong *et al*. 2000; Gibson *et al*. 2007). Crucially, this polymorphism is not used to describe the mitotypes (Fig. 2b), is not present in the 50 most common sequences used for the phylogeny (Fig. 2a) and therefore was not included in any of the mitotype counts (Figs 3, 4, 5, 6). Despite this, further work needs to be carried out to determine the validity of mitotypes as a proxy for pathotypes and indeed if suitable causal polymorphisms can be identified. Thus, we describe the distribution of mitotypes, with reference to the pathotype system, until further evidence in favour is acquired. Nevertheless, cyt B mitotypes provide an ideal proof of concept for the approach, which can be readily re‐adopted to any genetic sequence for cyst nematodes, or indeed any other organism of interest, for which suitable samples can be acquired or are available.

We expected the adopted approach could identify novel mitotypes which represent either new introductions or new mutations. Sequence 7 lies above our stringent sensitivity and specificity criteria and therefore may represent a novel mutation/introduction rather than resulting from an artefact of the approach. Errors in the approach are likely to be randomly distributed across all samples rather than being highly localized. Nevertheless, the identified field, and adjacent fields, should form the basis of future sampling. Re‐identification in an independent experiment would be testament to the validity and will inform distribution/spread.

The data presented here suggest that all 3 mitotypes are more widely and evenly distributed across Scotland than previously thought, particularly for mitotype 1 (Hockland *et al*. 2012). Given that the majority of fields contain only a single mitotype, the approach described here has the potential to provide recommendations as to appropriate cultivar choice on a per‐field basis. However, multiple introductions of cysts into the same field, even separated by decades, can result in highly heterogeneous population structures due to the long dormancy time. We demonstrate that over one‐fifth of all fields contain a mix of at least two mitotypes. In these cases, and in particular those containing all three mitotypes, cultivar recommendations will remain challenging. The discovery that certain combinations of mitotypes co‐occur more frequently than others is surprising. This may result from frequent co‐introductions of these populations or reflect the lack of discrete boundaries between true pathotypes (Phillips & Trudgill 1998).

Despite regular ploughing, mixed fields are dominated by local peaks of single mitotypes. This discovery presents an interesting opportunity to test the selection pressures imposed by various cultivars on the pathoscape and indeed critical evaluation of mitotypes as a proxy for pathotypes. The field can be planted, resampled and re‐assessed to determine effect of cultivar choice, or indeed nematicide application, on the population composition. Given the accuracy with which we are able to distinguish between narrow ratios of mitotypes (Fig. 3), even fine perturbations from a single cropping cycle should be detectable. Regularly sampling the same fields across a country will allow analysis of the spread or decline of certain mitotypes over time. This is particularly poignant for the study of putative novel mutations/introductions, as described above, which may increase in prevalence. The risks associated with emergence and subsequent spread of new highly virulent lineages of the potato late blight *Phytophthora infestans* are well recognized (Chowdappa *et al*. 2013).

It has been suggested that genetic variation at the level of field, or indeed region, is already described at the level of the individual host plant (Plantard *et al*. 2008). Given that (i) all mitotypes have a broad national distribution and yet ~80% of fields have a single mitotype and (ii) within mixed fields, local troughs and peaks of single mitotypes dominate, the data presented indicate this suggestion underestimates the true detail of PCN genetic variation. Our study also provides evidence in favour of ‘hybrid’ cysts and identifies geographical locations where these are likely to occur (on the basis of the presence of mixed populations of PCN). If the original introductions of *G. pallida* from S. America were geographically isolated, as suggested in the study by Plantard *et al*. (2008), then multiple mitotypes within a field could give rise to novel hybrids. Also given the polyandrous nature of the female potato cyst nematode, the implications for virulence of interpathotype breeding require investigation. Furthermore, the restricted inheritance of mitochondrial genomes may suggest that hybrid mitotypes are actually underestimated.

Errors can be introduced at every step in the described approach: barcode design, primer synthesis, PCR, library preparation, sequencing and bioinformatic analyses. While some error is unavoidable, error propagation throughout the experiment was of real concern, and thus at every stage, at least one measure was included to minimize error where possible. This, coupled with the stringent bioinformatic filtering and inclusion of appropriate controls, resulted in a useful level of sensitivity and specificity. The adopted approach was designed to, as far as possible, reduce costs and facilitate reproduction of the results. The aim is to promote adoption of this technique in nematology and further afield. The ability to simultaneously address multiple high‐value questions in a single experiment is testament to the power of next‐generation sequencing.

S.E.V.D.A., V.C.B. and C.J.L. designed research. S.E.V.D.A. performed research and analyzed the data. A.R., E.A., J.P., M.B., P.E.U. and J.T.J. contributed regents or analytical tools. S.E.V.D.A. and V.C.B. wrote the paper.

## Supporting information


**Table S1** Primers which amplify a region of *G. pallida* cyt B that is descriptive of each mitotype.
**Table S2** Thirty, 4 base pair barcodes, designed to be unique by at least two base pairs.
**Table S3** SNP comparison between mitotypes 1, 2, 3 and the 7^th^ most common sequence**.**

**Fig. S1** Mapping of the distribution of mitotypes at an additional site within a mixed field supports complex ‘Pathoscapes’.
