# Supplementary material for: A metagenetic approach to determine the diversity and distribution of cyst nematodes at the level of the country, the field and the individual
Source: Mol Ecol. 2015 Nov 26;24(23):5842–51. doi: 10.1111/mec.13434 (PMC4981918; doi:10.1111/mec.13434)
Supplement: Supplementary file 1 — Table S1 Primers which amplify a region of G. pallida cyt B that is descriptive of each mitotype. Table S2 Thirty, 4 base pair barcodes, designed to be unique by at least two base pairs. Table S3 SNP comparison between mitotypes 1, 2, 3 and the 7th most common sequence. [file MEC-24-5842-s001.docx]

| Forward primer name | Forward primer sequence | Reverse primer name | Reverse primer sequence |
| --- | --- | --- | --- |
| F1 | **CTTGAAGACCTTCTGTAAAAATG** | **R1** | **CGAGCTACCGTCTTAAGAG** |
|  |  | R2 | GTTTTTCCGGCTTCTTC |
|  |  | R3 | GTGAATGAGGGTTGGTTTTTCC |
|  |  | R4 | GGTTTTCTATTACTCTAATTCTTC |

**Table S1: Primers which amplify a region of *G. pallida* CytB that is descriptive of each type.** Bold indicates primer pair with most efficient amplification for low template concentrations. With respect to GenBank DQ631912.1, the region amplified is 281-590.

| Barcode number | sequence | Barcode number | sequence |
| --- | --- | --- | --- |
| 1 | TTTT | 16 | GAGA |
| 2 | TTAA | 17 | AGAG |
| 3 | AATT | 18 | CGCG |
| 4 | GGTT | 19 | GCGC |
| 5 | TTGG | 20 | ATGC |
| 6 | CCTT | 21 | TAGC |
| 7 | TTCC | 22 | GCTA |
| 8 | ATAT | 23 | GCAT |
| 9 | TATA | 24 | TACG |
| 10 | GTGT | 25 | ATCG |
| 11 | TGTG | 26 | CGTA |
| 12 | CTCT | 27 | CGAT |
| 13 | TCTC | 28 | CTAG |
| 14 | CACA | 29 | TCAG |
| 15 | ACAC | 30 | AGCT |

**Table S2:** Thirty, 4 base pair barcodes, designed to be unique by at least two base pairs.

| Position | 61 | 118 | 137 | 158 | 228 |
| --- | --- | --- | --- | --- | --- |
| Mitotype 1 | G | A | A | A | T |
| Mitotype 2 | G | G | A | A | C |
| Mitotype 3 | A | G | G | C | T |
| 7th seq | G | G | G | C | T |

**Table S3**: SNP comparison between mitotypes 1, 2, 3 and the 7^th^ most common sequence
